# Depressive symptoms in a European comparison – Results from the European Health Interview Survey (EHIS) 2

**DOI:** 10.25646/6227

**Published:** 2019-12-11

**Authors:** Ulfert Hapke, Caroline Cohrdes, Julia Nübel

**Affiliations:** Robert Koch Institute, Berlin Department of Epidemiology and Health Monitoring

**Keywords:** DEPRESSIVE SYMPTOMS, DEPRESSIVE SYMPTOM SEVERITY, SEX AND AGE, EUROPEAN COMPARISON

## Abstract

Depression is associated with a significant individual and social burden of disease. The European Health Interview Survey (EHIS) provides data that can be used to compare the situation in Germany to that of other European countries. Data was evaluated from 254,510 interviewees from Germany and 24 additional Member States of the European Union (EU). Depressive symptoms as defined by the Patient Health Questionnaire (PHQ-8) were used as an indicator of depression. The prevalence in Germany (9.2%) is higher than the European average (6.6%). However, when the severity of depression is taken into account, only the prevalence of mild depressive symptoms is higher (6.3% versus 4.1%). In Germany, young people display depressive symptoms more frequently (11.5% versus 5.2%) than older people (6.7% versus 9.1%). These results should be discussed against the backdrop of differences in age and social structure and point toward a need for prevention and provision of care targeting younger people in Germany, in particular.

## Introduction

Depression is associated with a significant individual and social burden of disease [1-4]. A reduction in quality of life and productivity are not only observed in individuals with manifest depression, but also in people with depressive symptoms [1, 5]. Although depression has become a focus of national and European public health measures, an actual comparison of the prevalence of depression in Germany with the other European countries is scarse [6]. Aiming at developing possible approaches for targeted national and European public health activities that go beyond the country-specific prevalence of depressive symptoms, this article also considers sex and age differences and the severity of depressive symptoms.

## Indicator

In the European Health Interview Survey (EHIS), all Member States of the European Union (EU) collect data on health, healthcare, health determinants and the socioeconomic situation of their populations (Info box). The target group are people aged at least 15 years living in private households. A manual containing recommendations and guidelines on methodology and data collection is available to ensure a high degree of harmonisation among survey results [7]. Data for the second wave of EHIS (EHIS 2) was collected between 2013 and 2015 and, on average, took eight months. During the EHIS 2 survey period, the EU had 28 Member States. The EHIS quality report [8] and the article by Hintzpeter et al. [9] in this issue present the methodology of EHIS 2 in more detail.

**GEDA 2014/2015-EHIS**

**(for international comparisons)**

**Data holder:** Robert Koch Institute

**Aims:** To provide reliable information about the population’s health status, health behaviour and health care in Germany, with the possibility of a European comparison

**Method:** Questionnaires completed on paper or online

**Population:** People aged 15 years and above with permanent residency in Germany

**Sampling:** Registry office sample; randomly selected individuals from 301 communities in Germany were invited to participate

**Participants:** 24,824 people (13,568 women, 11,256 men)

**Response rate:** 27.6%

**Study period:** November 2014 - July 2015

More information in German is available at www.geda-studie.de and Lange et al. 2017 [11]

In Germany, EHIS is carried out as part of health monitoring at the Robert Koch Institute. EHIS 2 was integrated into the German Health Update (GEDA 2014/2015-EHIS) [10, 11]. A detailed description of the methodology applied in GEDA 2014/2015-EHIS can be found in Lange et al. [11].

Depressive symptoms were assessed using a country-specific version of the internationally established 8-item Patient Health Questionnaire (PHQ-8) [12]. The PHQ-8 comprises symptoms of a major depression during the last two weeks in line with the Diagnostic and Statistical Manual of Mental Disorders (DSM-IV, 4^th^ edition [13]): depressed mood, diminished interest, significant weight loss or poor appetite, insomnia or hypersomnia, psychomotor agitation or retardation, fatigue or loss of energy, feelings of worthlessness or excessive or inappropriate guilt, diminished ability to think or concentrate. Each of these items was rated on a scale ranging from 0 (not at all), 1 (on individual days), 2 (more than half of the days) to 3 (nearly every day). Answers are summarized to a sum score and values greater 10 indicate a depressive symptomatology. Whereas values between 10 and 14 indicate a ‘mild’ depressive symptomatology, values greater 14 indicate a ‘moderate to severe’ depressive symptomatology [12].

The findings on depressive symptoms are based on the answers provided by 254,510 participants (139,614 women, 114,896 men) in the age groups 15 to 29 years, 30 to 44 years, 45 to 64 years and ≥ 65 years. Twenty-five out of the 28 EU Member States (excluding Belgium, the Netherlands and Spain) provided valid data.

Prevalences are stratified by sex and EU Member State. The precision of prevalences can be estimated based on 95% confidence intervals (95% CI). A wide 95% CI indicates greater statistical uncertainty of the results. A statistically significant difference between groups can be assumed if the corresponding p-value is smaller than 0.05. Differences between the EU average and the individual EU Member States were assessed using regression analyses (with Germany as the reference category). The analyses differentiated between (1) the prevalence of depressive symptoms and (2) the severity of symptoms. To control for systematic differences between EU Member States, analyses were performed in control of age, sex, education and income status and, moreover, was the clustering of individual data within each Member State taken into account.

**Figure 1:**
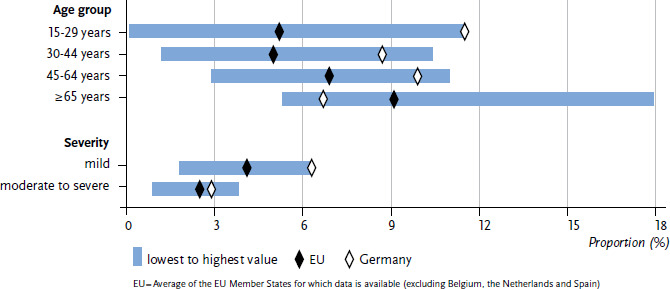
Age standardised prevalence of depressive symptoms during the last two weeks by age and severity (mild depressive symptoms: PHQ-8 10-14 points; moderate to severe depressive symptoms: PHQ-8 >14 points) Source: EHIS 2 (2013-2015)

In order to present the indicators more clearly, Figure 1 does not provide individual values for each EU Member State; instead, it describes the minimum and maximum values from the EU countries that provide data. Figure 1 also displays the average for the included EU Member States and the prevalence in Germany.

The analyses applied a weighting factor to account for each EU Member State proportionally according to the size of its population. In line with the recommendations by Eurostat, education was not used as a weighting factor for the comparison of European countries [11]. This leads to differences from previously reported German prevalences based on data from GEDA 2014/2015-EHIS [14]. In order to enable greater comparability of health indicators, values are standardised by age and sex in accordance with the revised European standard population (ESP) for 2013. This corrects for possible differences between the age structures found in the various countries and, therefore, enhances the comparability of health indicators [15]. The following analyses used the household indicator variable as a cluster variable.

**Info box**

**European Health Interview Survey (EHIS)**

The European Core Health Indicators (ECHI) were jointly developed by EU Member States and international organisations, taking into account scientific and health policy requirements. The indicators provide a framework in European health reporting for population-based health surveys and analyses, and health care provision at the European and national level. The European Health Interview Survey (EHIS) is a key element in this regard. The first EHIS wave (EHIS 1), which was not mandatory, was conducted between 2006 and 2009. 17 Member States and two non-EU countries participated in EHIS 1. Participation in the second wave of EHIS (EHIS 2), which was conducted between 2013 and 2015 in all EU Member States (as well as in Iceland, Norway and Turkey) was legally binding and is based on Commission Regulation (EU) No 141/2013 of 19 February 2013. It provides essential information about the ECHI indicators. In Germany, EHIS is carried out as part of health monitoring at the Robert Koch Institute. During the EHIS 2 survey period, the EU had 28 Member States.

Further information is available at: https://ec.europa.eu/eurostat/web/microdata/european-health-interview-survey

## Results and discussion

The prevalence of depressive symptoms in Germany (9.2%) is higher than the European average (6.6%) and higher than in most EU Member States, with the exceptions of Luxemburg, Sweden and Portugal (Table 1).

In the majority of EU Member States, women are more frequently affected by depressive symptoms than men. In Germany, 10.8% of women show depressive symptoms while the prevalence of men is considerably lower with 7.6% (Table 1). Moreover, the average prevalence across the EU for both women (7.9%) and men (5.2%) is lower than in Germany. Prevalences did not differ significantly between women and men in Finland, Ireland, Croatia, Luxemburg, Romania, Slovakia, Austria and the Czech Republic (Table 1).

In Germany, as well as in other EU Member States such as Ireland, Luxemburg and Sweden, adolescents and young adults (15- to 29-year-olds) show the highest prevalence of depressive symptoms. In Germany, the prevalence in this age group (11.5%) is significantly higher than the EU average (5.2%) (Figure 1). In other EU Member States such as Italy, Portugal and Romania, the prevalence is the highest among persons aged 65 and older (11.6%, 14.7% and 13.9%, respectively). The prevalence in this oldest age group (6.7%) is lower in Germany as compared to the EU average (9.1%).

As shown in Figure 1, the prevalence of mild depressive symptoms is higher in Germany (6.3%) than the EU average (4.1%) and represents the European peak value. Only Luxemburg has a comparably high prevalence (6.1%). Regarding the prevalence of moderate to severe depressive symptoms, Germany (2.9%) is close to the EU average (2.5%). Higher prevalences than in Germany can be found in Bulgaria (3.5%), Luxemburg (3.8%), Portugal (3.2%), Hungary (3.0%) and the United Kingdom (3.3%). However, these differences are not statistically significant.

The Europe-wide collection of PHQ-8 data in EHIS 2 enables a simultaneous comparison of the prevalence of depressive symptoms among individuals covering the adult life span for the first time. The results for Germany indicate a particularly high prevalence of depressive symptoms. In addition, findings from other German national surveys suggest an increase of depressive symptoms [14] and the risk of depression faced by younger women, as well as depression-related impairments [16] over time. The increasing importance of depression is also substantiated by data from German healthcare provision [17]. The present results point toward a particular need for public health action in terms of prevention measures and provision of care in Germany. Thereby, the risk of developing manifest depressive disorders could be reduced.

Strengthening prevention and the treatment of depression has been a national health target since 2006 [18]. In Germany, working environments are considered as one starting point for prevention measures addressing (mild) depressive symptoms [19]. Following a decision by Germany’s Federal Labour Court in 2008, the ‘mental and psychological integrity of workers and employees’ became a criterion in workplace hazard assessments (12 August 2008, 9 AZR (case number for the appeal) 1117/06). Since the embodiment of paragraph 5 point 6 of the Occupational Safety and Health Act, ‘psychological stress at work’, in 2013, the prevention of mental distress in workplaces and the consideration of mental health has gained more attention as a transversal issue in several social areas. Accordingly, the European Joint Action for Mental Health and Wellbeing defined ‘Mental Health in all Policies’ and ‘Mental Health at Workplaces’ as two out of five action fields for intervention.

However, when stratified by severity, the differences between Germany and the EU average only apply to mild depressive symptoms. One possible explanation, beyond potential differences in morbidity, refers to differences in health competence regarding mental well-being (‘mental health literacy’ [20]). Varying levels of mental health literacy are associated with differences in willingness to report mental health symptoms, and, therefore, can influence the responses that participants provide in the respective countries [21-23]. Increasing knowledge and improved understanding of the symptoms of mental health problems in the population may also lead to greater sensitivity towards depressive symptoms [22].

Furthermore, the present results indicate that in Germany – as well as in most of the other EU Member States – women are affected more frequently by depressive symptoms than men. This difference between the sexes is consistent with other international results [24, 25]. Beside biological factors, the higher prevalence of women is currently discussed in terms of cumulating psycho-social stressors.

In addition, particularly German young adults show a higher prevalence of depressive symptoms than the EU average. Results are also in line with previous findings of a higher prevalence of depressive symptoms among older adults in southern European nations such as Italy, Portugal and Romania as compared to Germany [26]. Possible explanations include regional differences in social structure such as education, income and unemployment rate [27, 28], health care availability, for example an ‘overdiagnosis’ of elderly people [29], and cultural differences such as (self)stigmatisation [30]. Future surveys also need to consider possible differences in data collection methodology [8].

At the national level, differences in the frequency of depressive symptoms have already been discussed against the backdrop of a region’s age and social structure, the spatial distribution of risk and protective factors, as well as the degree of urbanisation [14]. However, the reason why especially young adults show such a high prevalence in Germany and the health policy measures and contexts that could or should be used to reach them, remains an open question – particularly, because healthcare services in Germany identified depression rather often among the elderly [31].

Finally, it is also important to emphasise that the results based on the PHQ symptom questionnaire have to be interpreted as one possible indicator of the prevalence of depressive symptoms. In addition, (mild) depressive symptoms cannot be equated with a diagnosis of depressive disorder; consequently, it is not possible to draw valid conclusions about the (subjective) need for treatment. A differentiated comparison of mental health requires the comprehensive surveillance of multiple indicators and data sources at the national and European level.

## Key statements

- The prevalence of depressive symptoms in Germany is higher (9.2%) than the European average (6.6%).
- Significant differences between Germany and the EU average pertain to the prevalence of mild, but not moderate or severe depressive symptoms.
- Equally to the majority of other EU Member States, depressive symptoms in Germany are more frequent among women as compared to men.
- Depressive symptoms are more frequent among young people in Germany than the EU average (11.5% compared to 5.2%) and less frequent among older people than the EU average (6.7% compared to 9.1%).

## Figures and Tables

**Table 1:** Age standardised prevalence of depressive symptoms (PHQ-8 ≥ 10 points) during the last two weeks by sex and EU Member State (n=139,614 women, n=114,896 men) Source: EHIS 2 (2013-2015)

Member State	Women		Men		Total	
	%	(95% CI)	%	(95% CI)	%	(95% CI)
Austria	5.1	(4.5-5.8)	3.4	(2.8-4.2)	4.3	(3.8-4.8)
Bulgaria	8.0	(7.1-9.1)	6.0	(5.1-7.1)	7.1	(6.3-7.9)
Croatia	3.4	(2.8-4.1)	3.4	(2.8-4.3)	3.4	(2.9-4.0)
Cyprus	5.2	(4.3-6.3)	3.0	(2.3-3.9)	4.1	(3.5-4.9)
Czech Republic	3.4	(2.8-4.1)	2.0	(1.5-2.7)	2.7	(2.3-3.2)
Denmark	9.5	(8.4-10.6)	5.3	(4.4-6.3)	7.4	(6.7-8.2)
Estonia	8.0	(7.0-9.0)	5.0	(4.1-6.0)	6.6	(5.9-7.3)
Finland	6.4	(5.6-7.3)	5.7	(4.8-6.8)	6.0	(5.4-6.7)
France	9.0	(8.3-9.8)	5.2	(4.6-5.8)	7.2	(6.7-7.7)
**Germany^1^**	**10.8**	**(10.2-11.4)**	**7.6**	**(7.1-8.2)**	**9.2**	**(8.8-9.6)**
Greece	3.8	(3.3-4.5)	2.5	(1.9-3.3)	3.2	(2.8-3.7)
Hungary	9.6	(8.7-10.7)	7.1	(6.1-8.3)	8.5	(7.7-9.3)
Ireland	8.8	(7.8-10.0)	6.6	(5.7-7.7)	7.8	(7.0-8.5)
Italy	5.6	(5.2-6.0)	3.5	(3.2-3.9)	4.6	(4.3-4.9)
Latvia	5.8	(5.0-6.6)	3.3	(2.7-4.1)	4.7	(4.2-5.3)
Lithuania	4.1	(3.5-4.8)	2.3	(1.7-3.1)	3.3	(2.9-3.8)
Luxemburg	11.7	(10.3-13.2)	8.2	(7.0-9.6)	10.0	(9.0-11.0)
Malta	4.4	(3.6-5.4)	2.2	(1.6-3.0)	3.3	(2.8-3.9)
Poland	5.5	(5.0-6.0)	4.0	(3.5-4.5)	4.8	(4.4-5.1)
Portugal	12.9	(11.9-13.9)	4.7	(4.1-5.4)	9.1	(8.5-9.7)
Romania	5.1	(4.6-5.6)	4.7	(4.2-5.4)	4.9	(4.5-5.3)
Slovakia	3.4	(2.8-4.2)	2.3	(1.7-3.1)	2.9	(2.4-3.4)
Slovenia	7.3	(6.4-8.2)	4.0	(3.2-4.8)	5.6	(5.1-6.3)
Sweden	11.2	(10.0-12.4)	6.5	(5.7-7.4)	8.8	(8.1-9.6)
United Kingdom	8.6	(7.9-9.3)	6.1	(5.5-6.8)	7.4	(6.9-7.9)
**EU**	**7.9**	**(7.7-8.1)**	**5.2**	**(5.1-5.4)**	**6.6**	**(6.5-6.8)**

^1^ Statistically significant differences: total Germany vs. EU (p < 0.01), women Germany vs. EU (p < 0.01), men Germany vs. EU (p < 0.01)

CI = Confidence interval, EU = Average of the EU Member States for which data is available (excluding Belgium, the Netherlands and Spain)
